# Topographic Clinical Insights From Deep Learning–Based Geographic Atrophy Progression Prediction

**DOI:** 10.1167/tvst.13.8.6

**Published:** 2024-08-05

**Authors:** Julia Cluceru, Neha Anegondi, Simon S. Gao, Aaron Y. Lee, Eleonora M. Lad, Usha Chakravarthy, Qi Yang, Verena Steffen, Michel Friesenhahn, Christina Rabe, Daniela Ferrara

**Affiliations:** 1 Genentech, Inc., South San Francisco, CA, USA; 2 Roger and Angie Karalis Johnson Retina Center, University of Washington, Seattle, WA, USA; 3 Department of Ophthalmology, School of Medicine, University of Washington, Seattle, WA, USA; 4 Department of Ophthalmology, Duke University Medical Center, Durham, NC, USA; 5 Royal Victoria Hospital (The Belfast Trust), Queens University of Belfast, Belfast, UK

**Keywords:** ablation, age-related macular degeneration, artificial intelligence, deep learning, fundus autofluorescence, geographic atrophy

## Abstract

**Purpose:**

To explore the contributions of fundus autofluorescence (FAF) topographic imaging features to the performance of convolutional neural network–based deep learning (DL) algorithms in predicting geographic atrophy (GA) growth rate.

**Methods:**

Retrospective study with data from study eyes from three clinical trials (NCT02247479, NCT02247531, NCT02479386) in GA. The algorithm was initially trained with full FAF images, and its performance was considered benchmark. Ablation experiments investigated the contribution of imaging features to the performance of the algorithms. Three FAF image regions were defined relative to GA: Lesion, Rim, and Background. For No Lesion, No Rim, and No Background datasets, a single region of interest was removed at a time. For Lesion, Rim, and Background Shuffled datasets, individual region pixels were randomly shuffled. For Lesion, Rim, and Background Mask datasets, masks of the regions were used. A Convex Hull dataset was generated to evaluate the importance of lesion size. Squared Pearson correlation (*r*^2^) was used to compare the predictive performance of ablated datasets relative to the benchmark.

**Results:**

The Rim region influenced *r*^2^ more than the other two regions in all experiments, indicating the most relevant contribution of this region to the performance of the algorithms. In addition, similar performance was observed for all regions when pixels were shuffled or only a mask was used, indicating intensity information was not independently informative without textural context.

**Conclusions:**

These ablation experiments enabled topographic clinical insights on FAF images from a DL-based GA progression prediction algorithm.

**Translational Relevance:**

Results from this study may lead to new insights on GA progression prediction.

## Introduction

Geographic atrophy (GA) is an advanced stage of age-related macular degeneration (AMD), characterized by the progressive loss of photoreceptors, retinal pigment epithelium (RPE), and choriocapillaris that affects approximately 20 million individuals in the United States.^1^ Fundus autofluorescence (FAF) is one of the retinal imaging modalities of choice in patients with GA because it documents intrinsic fluorophores within lipofuscin granules in the RPE, allowing topographic mapping of this cellular layer.^2^ The change in GA lesion area as measured on FAF over a defined period of time (i.e., the GA growth rate) has been broadly utilized as the primary endpoint in clinical trials for this condition.^3^^–^^5^ In turn, the ability to predict the GA growth rate of individual patients can enhance clinical trial design in a variety of very useful ways, including covariate adjustment during the primary analysis of treatment effect to increase the power of the trial.^6^^–^^9^ The ability to predict GA growth rate can also be informative for patient counseling and treatment decisions. Therefore, accurate prognostic models of GA growth rate have relevant utility for drug development and clinical practice.

There is large variability in GA growth rate between individuals, of which approximately 50% can be explained by GA progression prediction models.^10^^,^^11^ Several studies have investigated both retinal imaging features and clinical features that are predictive of GA growth rates,^12^^–^^14^ including but not limited to GA lesion perimeter as measured by color fundus photography^15^; GA shape-descriptive features and surrounding abnormal autofluorescence patterns as measured on FAF^16^^,^^17^; the presence of reticular pseudodrusen as measured on FAF and near-infrared (NIR) imaging^18^; the presence of outer retinal tubulation as measured on optical coherence tomography (OCT)^19^; choriocapillaris flow void as measured on OCT angiography^20^; and genetic, environmental, and demographic factors.^21^ The previous GA growth rate of an individual eye was also found to be an important predictor of future GA growth rate of that eye.^22^ Despite this body of knowledge, the exact pathological mechanisms underlying GA lesion onset and progression remain unknown.^23^ Not surprisingly, prognostic models that self-select relevant retinal imaging features, such as convolutional neural network (CNN)-based deep learning (DL) algorithms, have the potential to deliver improved performance compared with human-selected, feature-based studies.^24^^,^^25^ However, the tradeoff of using this approach is that the features driving the performance of DL models remain unknown, which limits the interpretability of their outputs.^24^

The goal of this study, therefore, was to characterize the specific imaging regions and features that drive the success of CNN-based DL algorithms predicting the GA growth rate. This is valuable for several reasons. First, it has the potential to provide new insights into the pathophysiology of the disease. Second, if specific image features are identified, they can be characterized, quantified, and used for building more interpretable models that predict GA growth rate.

There are many ways to characterize features that drive the success of imaging-based DL algorithms.^26^^–^^28^ Among these options, ablation of the imaging data using a remove-and-retrain strategy is best suited to our purpose, because, although computationally expensive, it allows for the most direct examination of the relative contributions of regions of interest while retaining the same distribution between development and holdout sets.^29^

In this study, we utilized this technique to selectively explore the contributions of specific regions of the FAF image in eyes with GA secondary to AMD and the imaging features within those regions. Specifically, we investigated three regions: the area of the GA lesion, a 500-µm rim surrounding the GA lesion, and the macular area beyond the rim encompassed within the image background. We also used intensity shuffling methods to explore the contribution of FAF pixel intensity values and textural attributes. In doing so, we aimed to identify the topographic regions and FAF signals of greatest relevance for a DL-based prediction of GA progression.

## Methods

### Datasets

This retrospective study used data from the study eyes of patients with bilateral GA enrolled in the lampalizumab phase 3 clinical trials (Chroma, NCT02247479; Spectri, NCT02247531) and in an accompanying observational study (Proxima A, NCT02479386). The study eye inclusion criteria in these trials were previously described.^3^^,^^4^ Briefly, the inclusion criteria were bilateral GA, total GA area of 1 to 7 disc areas in the study eye, FAF pattern banded or diffuse in the study eye, and absence of past or current choroidal neovascularization in either eye. The trials adhered to the tenets of the Declaration of Helsinki and were Health Insurance Portability and Accountability Act compliant. Protocols were approved by the institutional review board at all participating sites prior to enrollment. All patients provided written informed consent for future medical research and analyses. Only images acquired at the screening visit were used in the prediction of GA growth and therefore unaffected by treatment status. In addition, no treatment effect was observed in the phase 3 trials.^4^ Hence, all images were pooled for analysis, as in previous reports.^30^

The present study used macular-centered 30° FAF images (768 × 768 and 1536 × 1536 pixels) from the study eye collected at the baseline visit with the SPECTRALIS HRA (Heidelberg Engineering, Heidelberg, Germany). All images were resized to 384 × 384 pixels to reduce the computational load during training. NIR images were also obtained at the same baseline visit and were used as a supportive imaging modality for segmentation of the lesion masks (see the following Creating the Lesion and Rim Masks section).

As part of the clinical trial protocol, GA lesion area (mm^2^) had been graded on FAF images collected at all study visits in a central independent reading center using RegionFinder software (Heidelberg Engineering) by two trained expert image graders (grader 1, G1; grader 2, G2), with an adjudication process if necessary. GA growth rate (mm^2^/y) was derived from a linear model fitted using all available GA lesion area measurements for each study eye that underwent FAF imaging every 24 weeks over 2 years, as the GA growth rate has been generally reported to be linear.^31^^–^^33^

For eligible studies, qualified researchers may request access to individual patient-level clinical data through a data request platform. At the time of writing, this request platform is Vivli (https://vivli.org/ourmember/roche/). For up-to-date details on Roche’s Global Policy on the Sharing of Clinical Information and how to request access to related clinical study documents, see https://go.roche.com/data_sharing. Anonymized records for individual patients across more than one data source external to Roche cannot, and should not, be linked due to a potential increase in risk of patient re-identification.

### Creating the Lesion and Rim Masks

The GA Lesion Mask was obtained as the output of a segmentation algorithm described previously.^34^ Briefly, two DL algorithms were developed on a separate dataset (Proxima B) for GA lesion segmentation: (1) UNet to segment a GA lesion from FAF images, and (2) YNet to segment a GA lesion from both FAF and NIR images. YNet using NIR in combination with FAF proved to be slightly advantageous when the segmentation outputs were compared with expert graders (G1 and G2), with YNet Dice scores of 0.92 (G1) and 0.90 (G2) and UNet Dice scores of 0.90 (G1) and 0.88 (G2). Therefore, in this work, YNet was used for segmentation for lesions with NIR imaging available (approximately 95% of cases) and UNet was used to segment the remaining 5% without NIR imaging.

From the segmentation output, three FAF image regions were defined as regions of interest: *Lesion*, the area of the GA lesion; *Rim*, a 500-µm rim surrounding the GA lesion^16^; and *Background*, the rest of the FAF image field outside of those. The 500-µm rim was chosen based on the publication of Bearelly et al.,^16^ showing that hyperautofluorescence within a 500-µm–wide margin bordering GA lesions was significantly associated with an increased GA growth rate. Because the FAF images were acquired with a 30° capture at approximately 290 µm per degree, each 384-pixel edge corresponded to approximately 8700 µm. With this calculation, a 22-pixel (500-µm) border surrounding the Lesion Mask was created by first dilating the Lesion Mask using the OpenCV package function morphologyEx with an elliptical kernel followed by subtracting the Lesion Mask. The Background region was the remaining portion of the FAF image that was not included in the Lesion or Rim Masks.

### Ablation Experiments

**Figure 1.**
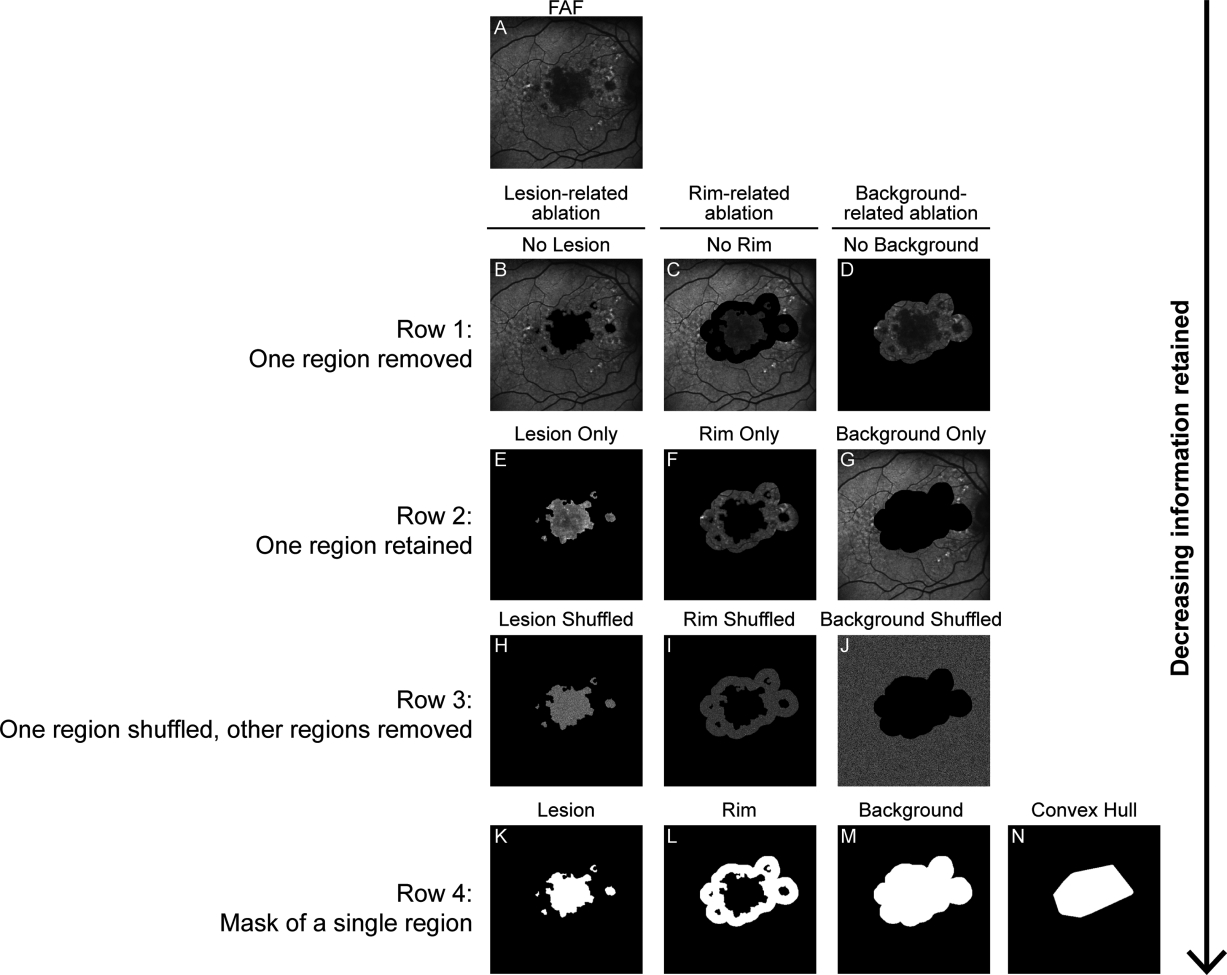
(**A**) A full FAF image is depicted as the reference for the ablated datasets (**B**–**N**). *Row 1* (**B**–**D**) depicts one region (Lesion, Rim, or Background) ablated with black pixels. *Row 2* (**E**–**G**) depicts two regions ablated, with one region retained at a time. *Row* 3 (**H**–**J**) depicts two regions ablated, with a single region retained and the pixels of that region randomly shuffled. *Row 4* (**K**–**M**) depicts the mask of a single region derived from the output of a previously described segmentation algorithm, and *Row 4* (**N**) depicts an example of a Convex Hull used to ablate additional shape features, such as focality and perimeter estimation. Lesion, inside the GA lesion; Rim, a 500-µm rim surrounding the GA lesion; Background, the region outside of those.

Figure 1A depicts an example of a full FAF image with no ablated regions as a reference image for visual comparison. The ablated datasets were initially created to investigate the contribution of the FAF texture features in the three regions of interest to the DL-based GA progression prediction algorithm. To do so, the algorithm was retrained with each ablated dataset, one at a time, and its performance was compared with the benchmark algorithm trained with the full FAF image dataset. The experiments that followed were designed to investigate the contribution of FAF signal intensity information and the contribution of features related to GA shape and size.

For the first three ablated datasets, each single region of interest was removed at a time and replaced with black pixels. Figure 1, row 1, depicts an example of an image with the GA lesion removed (No Lesion) (Fig. 1B), the GA rim removed (No Rim) (Fig. 1C), and the background removed (No Background) (Fig. 1D).

For the next three ablated datasets, two regions of interest were removed at a time while retaining one single region (Fig. 1, row 2): Lesion Only (Fig. 1E), Rim Only (Fig. 1F), and Background Only (Fig. 1G). These initial experiments assessed the three regions of interest while retaining their FAF texture features in certain regions, as the ablation experiments did not interfere with the pixels that were contained within each region.

The next experiments were designed to investigate the contribution of FAF signal intensity information relative to the contribution of the FAF texture features. Three new datasets were created, with pixels in one individual region of interest randomly shuffled and the other two regions ablated with black pixels, so the FAF signal intensity without texture in each individual region was investigated one at a time. Figure 1, row 3, depicts the Lesion Shuffled dataset (Fig. 1H), the Rim Shuffled dataset (Fig. 1I), and the Background Shuffled dataset (Fig. 1J).

Finally, to investigate the contribution of features related to GA shape and size, the masks of the regions themselves were used to interrogate the contribution of shape and size information (Fig. 1, row 4). The Lesion Mask dataset (Fig. 1K), the Rim Mask dataset (Fig. 1L), and the Background Mask dataset (Fig. 1M) are depicted in row 4. Finally, the Convex Hull dataset (Fig. 1N) was generated to evaluate how much information was being driven by relative size of the lesion versus additional shape and focality information included in the Lesion Mask dataset.

### Training Procedures

The dataset was split into the development (1041 patients) and holdout (255 patients) sets. Only one study eye image of each patient was used; therefore, no patient-level data leakage was possible. Baseline characteristics (including GA lesion area) of the eyes included in the analyses were well balanced across the dataset splits. The development set was further split into five folds for cross-validation (CV). This split was held constant for all ablated datasets.

A VGG16 network architecture was chosen as the base architecture for this experiment based on observed favorable performance on the full FAF image dataset (compared with other CNN architectures) and its favorable training time. The fixed hyperparameter choices for training include the following: the Adam optimizer, a OneCycle learning rate schedule, early stopping, and the mean squared error (MSE) loss function. A random grid search approach was used for data augmentation, dropout likelihoods, number of epochs trained, initial learning rate, and lambda (controlling weight decay). Supplementary Table S1 describes all possible choices for each hyperparameter; 30 configurations of these were randomly chosen and fixed for all ablation datasets to employ a random hyperparameter search strategy while still having fair comparisons between ablated datasets.^35^

With 13 different ways to ablate the images (13 ablated datasets) and one full FAF image dataset (14 total imaging datasets), 30 hyperparameter sets, and five models trained for each CV fold split, a total of 2100 VGG16 CNNs were trained. The VGG16 was pretrained on ImageNet obtained from the model zoo in PyTorch 1.9.0 (The Linux Foundation, San Francisco, CA). All training was done on Nvidia V100 and P6000 GPUs (Santa Clara, CA) (Supplementary Tables S1 and S2).

### Statistical Methods and Algorithm Performance Analysis

The square of the Pearson correlation (*r*^2^) between predicted and observed GA growth rates was used to measure the performance of the algorithms during fivefold CV.^7^ We chose *r*^2^ for two reasons: First, it is the performance metric that is directly translatable to our intended use of the prognostic model, which is a reduction in standard error. Second, when comparing models, we wanted to focus on discrimination ability, and *r*^2^ is less impacted by potential miscalibration of a model, whereas MSE or *R*^2^ could result in different ranking due to imperfect calibration (which would be difficult to assess with the large number of models trained for these experiments). Early stopping was used based on the performance on the validation fold. The *r*^2^ mean performance on the five outer folds was calculated, and the best model was chosen for each dataset based on the highest *r*^2^ mean. Each of the five models generated from those experiments was tested on the final holdout set for comparison. The highest *r*^2^ mean test performance from each dataset was compared to the benchmark *r*^2^ mean performance of the full FAF images.

## Results

### Contribution of FAF Texture Features in the Lesion, Rim, and Background FAF Regions

**Figure 2.**
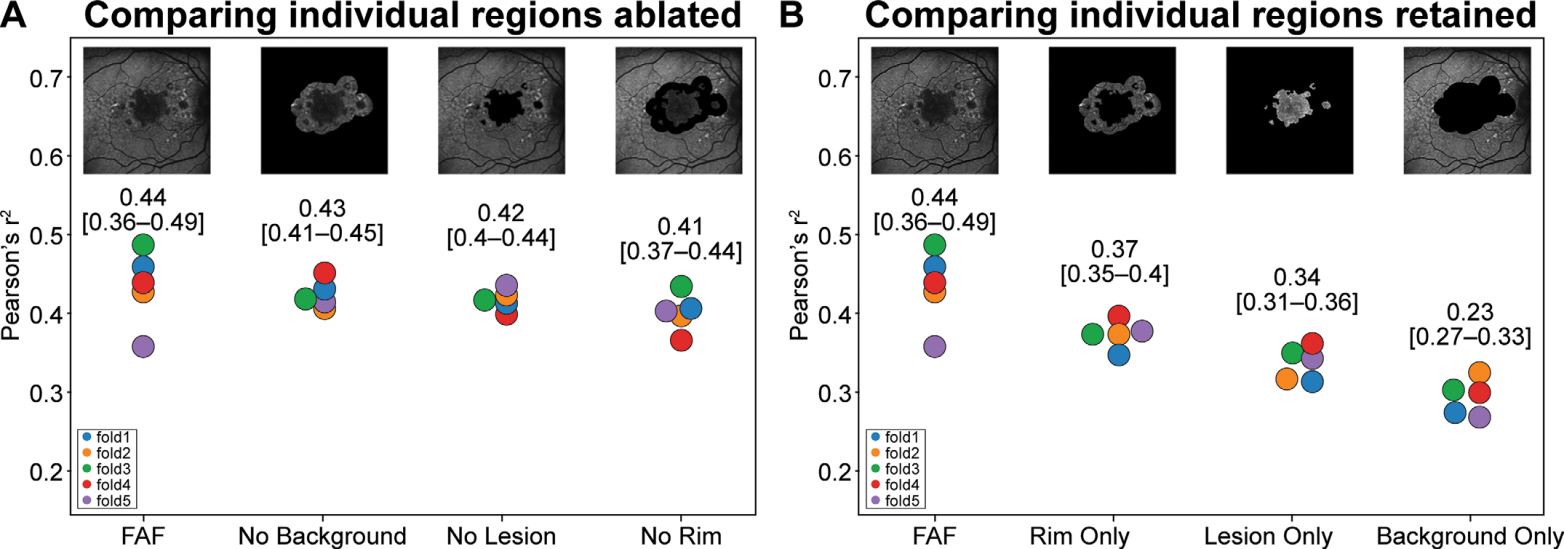
Contribution of FAF texture features of various ablation regions. For each experiment, the same hyperparameters were used to build five distinct DL models in a fivefold cross-validation experimental setup. The five trained models were then tested on the holdout set, and the performance (*r*^2^) was recorded as shown. (**A**) Comparison of the individually ablated regions (No Background, No Lesion, and No Rim) with the full FAF image. (**B**) Comparison of the individual regions retained (Rim Only, Lesion Only, and Background Only) with the full FAF image.

The performance of the algorithm predicting GA growth rate based on the full FAF image was found to have *r*^2^ mean of 0.44 and was taken as the benchmark to compare with all further experiments (Fig. 2A). The first set of experiments based on the ablation strategy removed a single FAF region at a time (No Background, No Lesion, and No Rim datasets) (Fig. 1, row 1) while preserving intact the pixels from the other two regions (Fig. 2A). These ablation exercises demonstrated that the No Background dataset achieved the next highest *r*^2^ mean at 0.43, whereas the No Lesion dataset achieved an *r*^2^ mean of 0.42 and the No Rim dataset achieved an *r*^2^ mean of 0.41.

The second set of experiments utilized only a single region at a time: the Lesion Only, Rim Only, and Background Only datasets (Fig. 2B). The performance was again compared to the benchmarked performance of the algorithm utilizing the full FAF image of *r*^2^ mean 0.44. The next best performance was observed utilizing the Rim Only dataset (*r*^2^ mean = 0.37), followed by the Lesion Only dataset (*r*^2^ mean = 0.34) and finally the Background Only dataset (*r*^2^ mean = 0.30). Both of the comparisons in Figures 2A and 2B reflect the same trend: The Rim contains the most important FAF texture information, followed by the Lesion, and then the Background.

### Contribution of FAF Signal Intensity Information

**Figure 3.**
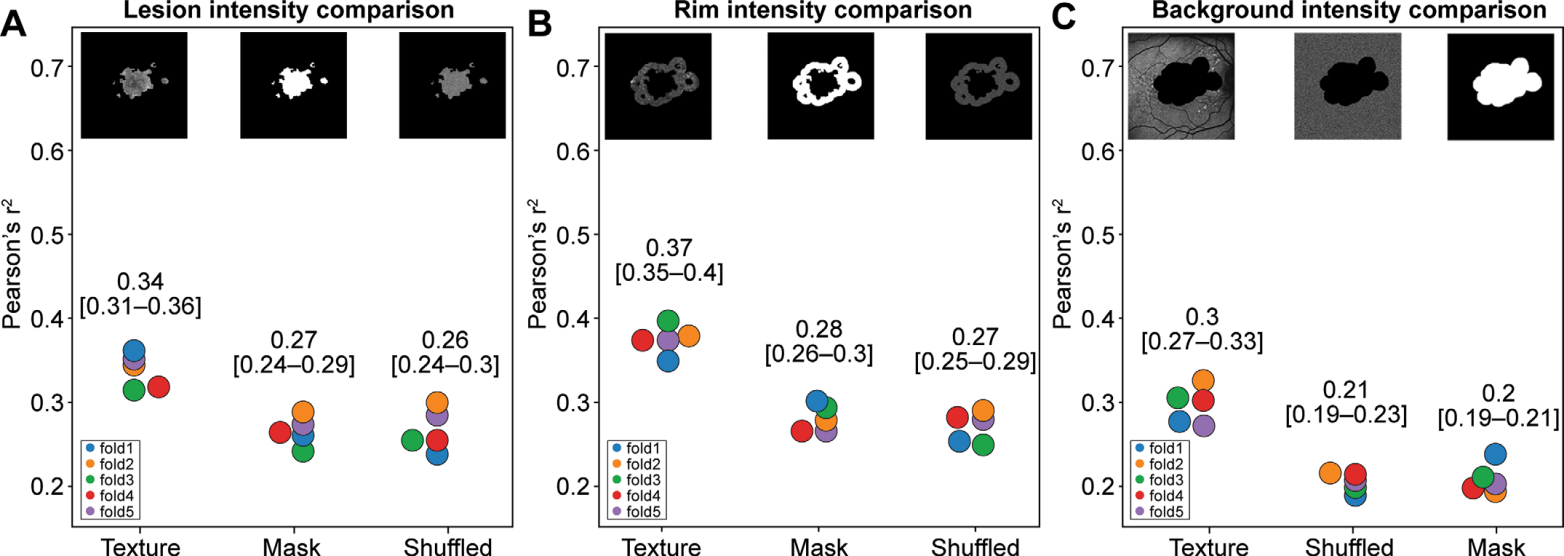
The contribution of FAF intensity information was investigated by shuffling the pixels in a given region of interest. Texture refers to one region retained with no alterations (retaining full texture information), Shuffled refers to the pixels within the region randomly shuffled to remove texture but retain intensity information, and Mask refers to the region mask only with no texture and no intensity information. Each panel refers to a separate region comparison: (**A**) Lesion, (**B**) rim, and (**C**) Background. In each case, the Mask and Shuffled datasets performed comparably.

The contribution of FAF signal intensity was assessed in each one of the three regions of interest by randomly shuffling the pixels within each region, so the FAF texture was lost but the FAF intensity signal remained in the images. The resultant *r*^2^ mean of each of these shuffled experiments (Fig. 1, row 3) was compared with datasets that retain texture (Lesion Only, Rim Only, and Background Only datasets) (Fig. 1, row 2), as well as those that remove pixel intensity entirely (the Lesion Mask, Rim Mask, and Background Mask datasets) (Fig. 1, row 4). Beginning with Lesion Only comparisons, Figure 3A shows that the algorithms trained with the Lesion Mask (*r*^2^ mean = 0.27) and the Lesion Shuffled (*r*^2^ mean = 0.26) datasets have nearly identical performance and much worse performance than the Lesion Only (*r*^2^ mean = 0.34) dataset retaining texture. Figures 3B and 3C show that this trend continues when comparing the performance of the algorithms trained with the Rim Only and Background Only datasets versus their respective Shuffled and Mask dataset variations.

### Contribution of Features Related to GA Shape and Size

**Figure 4.**
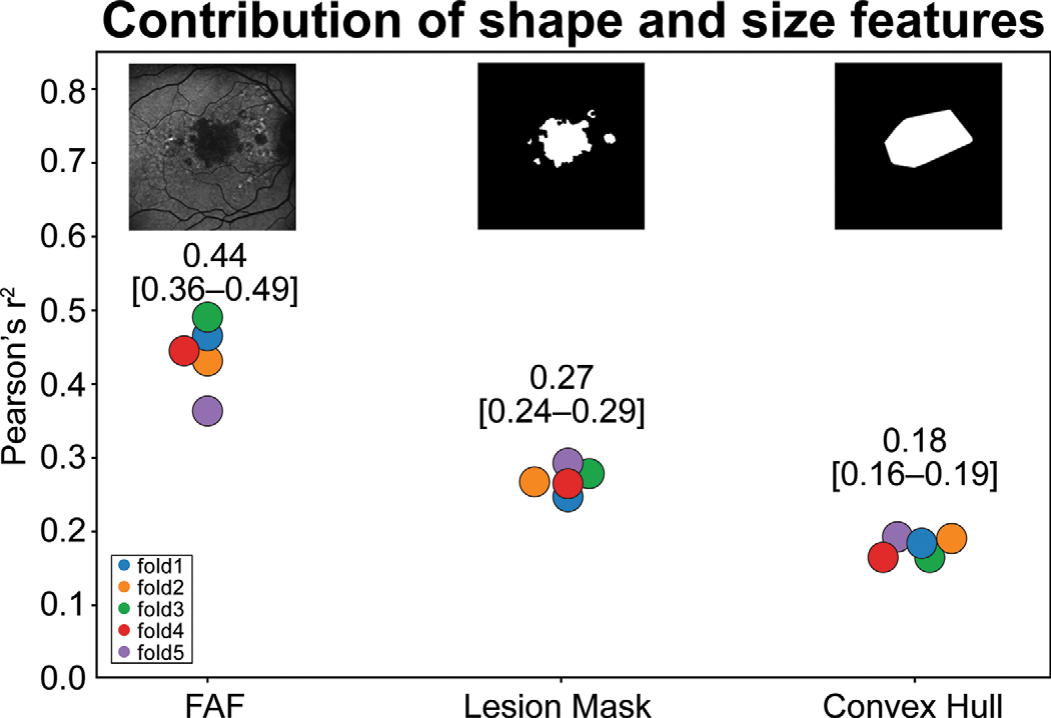
Contribution of GA features related to shape and size. In this figure, the full FAF image is used for reference and compared with the Lesion Mask (*r*^2^ mean = 0.27) and the Convex Hull (*r*^2^ mean = 0.18) to evaluate the contribution of GA features related to shape and size. Notably, GA focality, edge detail, and more precise area estimation account for the difference between 0.18 and 0.27, respectively; FAF texture features account for the 0.17 difference between 0.27 and 0.44, respectively.

The last set of experiments aimed to evaluate the contribution of features related to GA shape and size to the prediction of GA growth rate. The performance of algorithms trained with the Lesion Mask and Convex Hull datasets was again compared to the performance of the benchmarked algorithm trained with the full FAF image with an *r*^2^ mean of 0.44 (Fig. 4, Supplementary Fig. S1). The algorithm trained with the Lesion Mask dataset achieved a mean of 0.27 and that trained with the Convex Hull achieved an *r*^2^ mean of 0.18. In addition, a drop in performance was observed when comparing the Lesion Mask (*r*^2^ mean = 0.27) and Rim Mask (*r*^2^ mean = 0.28) datasets (which retain the perimeter and shape information) with the Background Mask (*r*^2^ mean = 0.2) dataset (which obscures the perimeter and shape information).

## Discussion

**Figure 5.**
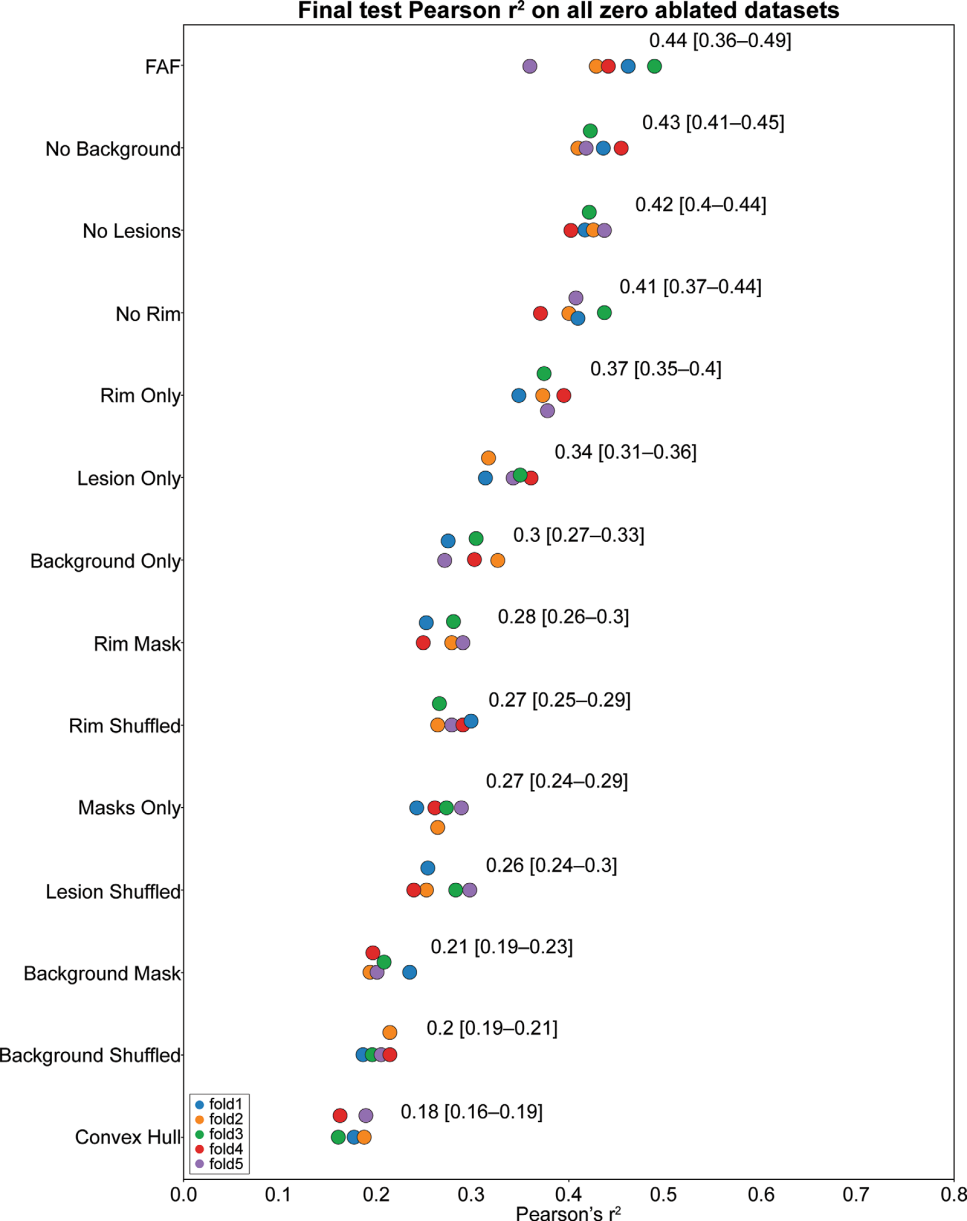
Comparison of the *r*^2^ results for all zero ablated datasets.

In this study, the contribution of various FAF imaging features to the DL prediction of future GA growth rate were investigated. In systematic experiments, image ablation experiments enabled a series of algorithm retraining exercises to investigate specific FAF image regions of interest relative to the GA lesion. The performance of an algorithm trained with the full FAF images was used as the benchmark. The changes in test *r*^2^ means on ablated datasets were used to analyze the contributions of different imaging features to CNN performance. Figure 5 shows all 13 different ablation experiments, as well as the one full FAF image dataset (14 total imaging datasets).

Our results showed that the benchmarked algorithm trained with the full FAF image performed the best. Rim ablation that retained the FAF texture had the greatest influence in algorithm performance, resulting in a considerable drop in the correlation coefficient. However, ablating the image background beyond the Rim had almost no impact on model performance, indicating that the image region that encompasses the GA Lesion and surrounding Rim is sufficient for the algorithm to extract relevant predictive information related to future GA growth rate. By removing specific FAF regions at a time while still retaining FAF texture, we also observed that model performance was best when only the Rim area was retained. These sets of experiments (Figs. 2A, 2B) are complementary to one another. Taken together, one can interpret these findings as an indication that the rim area contains the FAF texture-based biomarkers that contribute most to the performance of the DL algorithm, compared with those biomarkers within the lesion and the background regions. These findings are aligned with our understanding of the GA pathogenesis, since within the lesion the retinal tissue has already been lost due to the disease but the rim contains the tissue that will be affected next by the disease, whereas anatomical changes secondary to GA away from the center of the macula will take longer to appear in the background region. This alignment between the DL-based prediction and the clinical understanding of disease progression can aid to interpretability and trustworthiness of these predictions.

In fact, a robust body of clinical research has previously indicated the importance of retinal imaging biomarkers located in the rim, typically referred to as the junctional zone of GA on FAF and characterized by various patterns of relative increase or decrease of the FAF signal. In a few seminal papers from 2005 to 2007, Holz and collaborators^36^^,^^37^ defined phenotypic patterns in the junctional zone and characterized their differential prognostic value. Our rim ablation findings support previous literature reporting the prognostic value of the rim region, including the work by Holz et al., as well as by Bearelly et al.^16^ Briefly, four broad categories attempt to describe the junctional zone of GA on FAF: “normal” autofluorescence; “focal” hyperautofluorescence; “banded” hyperautofluorescence (increased FAF signal located at the Rim surrounding the entire perimeter of the GA border); and a “diffuse” pattern (increased FAF signal throughout the Rim and Background regions). The results from these previous studies indicate that the banded and diffuse FAF patterns are significantly associated with faster GA growth rates than focal or normal FAF patterns. Of note, only eyes with the banded and diffuse FAF patterns were included in the present study, according to the clinical trial eligibility criteria for the lampalizumab program. Thus, this study offers an opportunity to further explore more subtle prognostic imaging features within these phenotypes already known for their faster disease progression. Thus, the concordance on the relative clinical importance of these regions for disease progression in eyes with GA to both experts and the developed DL algorithms is of value and encourages a degree of trust in the findings from the DL models presented in this work.

After investigating the contribution of specific macular regions in the prediction of the GA growth rate, we also sought to investigate the contribution of FAF intensity features by retaining and shuffling the pixels within individual regions as shown in Figure 1, row 3. These experiments were compared with the results of the experiments from Figure 1, rows 2 (unshuffled) and 4 (mask). When we designed these experiments, we hypothesized that Figure 1, row 2, results would have the highest performance because the FAF image texture would be preserved, and, comparatively, the shuffled regions in Figure 1, row 3, would have lower performance due to removal of the image texture that relates to FAF patterns seen clinically. The hypothesis that FAF signal intensity would not significantly contribute to the algorithm performance is aligned with the clinical understanding that FAF is a qualitative but not a quantitative exam. This hypothesis was indeed supported by our results (Fig. 3).

On the other hand, we also hypothesized that the experiments utilizing datasets with shuffled pixels would demonstrate higher algorithm performance than those utilizing the mask of the corresponding region, because the intensity of the FAF signal would be retained in the shuffled datasets but entirely lost in the masks. Intensity-based biomarkers as clinically described have been associated with GA progression rate in previous studies; for example, rim area focal hyperautofluorescence (RAFH) has been positively correlated with the GA progression rate.^38^^,^^39^ Surprisingly, Figure 3B shows that the Rim Shuffled dataset had a similar performance compared to the Rim Mask. This finding suggests that RAFH is not captured by the models when the pixels are shuffled, despite an intuitive belief that FAF signal intensity information would be retained. The results are similar for both the Lesion and Background comparisons (Figs. 3A, 3C). One possible explanation is the texture bias in CNNs,^40^^,^^41^ especially those pretrained on ImageNet. Although there are steps outlined in Geirhos et al.^40^ to increase shape bias, there is no mention of how first-order intensity features can be taken into account for CNNs, despite evidence that they contribute to predictive performance. Nonetheless, taken together, these results are in line with the qualitative nature of FAF imaging, where FAF patterns are determined by the relative brightness of any given pixels in comparison to the surrounding pixels. In addition, no accurate measurements can be extracted from the FAF signal intensity based on the commercially available technology. For this reason, between-image intensities cannot be quantitatively compared by clinical expert graders. As an attempt to overcome the qualitative nature of FAF imaging, several groups have previously explored clinical research utilizing quantitative FAF analysis.^42^^-^^44^ Although some observations based on quantitative FAF may be intriguing, its overall interpretation has been somewhat inconclusive and poorly reproducible, with fundamental limiting factors that have been precluding its broad clinical utilization so far.^45^

Finally, the performance of the algorithms utilizing the Lesion and Convex Hull datasets was compared with the performance of the benchmarked algorithm utilizing the full FAF image to evaluate the contribution of imaging features related to GA shape and size (Fig. 4). Supplementary Figure S1 shows 10 randomly chosen patients and depicts their full FAF image, the Lesion Mask created for that image, and the Convex Hull for that mask. These were generated to illustrate the imaging information retained in the Convex Hull dataset; mainly, it removes focality information and all finer edge detail that is otherwise retained in the Lesion Mask dataset. Effectively, this strategy removes imaging features that have been previously shown to be prognostic for the prediction of GA growth rate, such as detailed area estimation, perimeter information, and circularity.^46^ Our results support that this removal of prognostic information impacts the performance of the DL models, as we observe that the Convex Hull dataset had poor performance compared with the Lesion Mask dataset (Fig. 4). From this comparison, it is possible to infer the increase in the ability of the model to predict GA growth with the added information of area, perimeter, circularity, and focality (information in the Lesion Mask but not in the Convex Hull). In addition, we compared the full FAF image to the Lesion Mask dataset to observe the impact of all FAF texture features (regardless of location). The observed increase in performance from the Lesion Mask alone to the full FAF image suggests that it will not be possible to rely on GA shape-based features alone to achieve performance comparable to that of DL models built with full FAF images. Indeed, these results suggest that such a model will likely have to include derived FAF texture features to achieve comparable performance.

The high quality and large size of the imaging dataset, collected in the context of a pivotal clinical trial program, and the state-of-the-art systematic approach are strengths of this research. However, the imaging dataset was also subject to imaging inclusion criteria of the clinical trials; therefore, it only represents banded and diffuse FAF patterns, so all of this work is only applicable to said patterns.

In addition, imaging ablation as a strategy for feature attribution has a few key advantages over its most comparable method of feature attribution, occlusion.^47^ These two methods are very similar but hold one key difference in that occlusion reuses a model trained on full images and occludes regions during test time, whereas our ablation strategy retrains the model. As a result, the drop in algorithm performance due to removal of the imaging regions can be attributed directly to the imaging region rather than the test data being out of distribution.^48^ Ablation with retraining, although much more computationally intensive, isolates the impact of image alteration and is therefore more interpretable, which is a key strength of the methodology applied in this study.

One important limitation of this work is that all of the Lesion Masks were derived from a previously developed GA lesion segmentation algorithm.^49^ The gold standard for lesion mask creation in eyes with GA does not exist. It may require multimodal retinal imaging annotations by expert graders using both FAF and OCT combined, in addition to other supportive imaging modalities such as NIR, to identify the GA lesion boundaries accurately. It is also possible that the boundaries generated by the segmentation algorithm may not coincide with expert annotation in some cases. In fact, the imperfection of the lesion segmentation can be observed in Supplementary Figures S1B and S1E. Therefore, the experiments evaluating the contribution of features related to shape and size (Fig. 4) might underestimate the value of these features. Further variability may have been introduced by using a separate segmentation algorithm (UNet) for images without NIR (∼5%). Another limitation related to shape and size features is that we used a single rim size of 500 µm based on a previous publication; however, it would be useful to evaluate the impact of the size of the rim, and this is a candidate for future work.

By identifying the regions of the FAF macular imaging that are mostly relevant to predict future GA growth rate, our work may also lead to new insights related to the pathobiology of this disease, which is still poorly understood. It is important to note that further research is required to accurately identify the FAF imaging features of the greatest prognostic importance. For example, we found that the Masks Only dataset performed better than the Convex Hull dataset (*r*^2^ mean = 0.18 and *r*^2^ mean = 0.27, respectively). However, we were unable to define how much of that performance gain was due to focality features such as number of lesions, a more accurate representation of area or circularity, or a more detailed perimeter shape and measurements. This is also true for the regional texture experiments (Fig. 3), where it is only possible to derive the relative importance of regions containing texture rather than the texture features per dataset.

In summary, our work has taken a rigorous approach to develop insight into the FAF imaging features driving the success of the ability of DL algorithms to predict future GA growth rates, a notoriously challenging task that cannot be performed by clinical experts. Our analysis supports previous quantitative and qualitative observations that the rim region (FAF junctional zone of GA) contains important prognostic texture information. In addition, our results will inform downstream imaging feature–based interpretable model development in a variety of important ways, because (1) we now have an approximation for the amount of variability explained from texture-based imaging features and shape-based imaging features; (2) we now know that intensity features may not yield much additional insight; and (3) during stepwise additive model building, we can prioritize texture features derived from the rim, followed by the lesion, and, finally, the background regions of FAF images.

## Supplementary Material

Supplement 1
