# Supplementary material for: Topographic Clinical Insights From Deep Learning–Based Geographic Atrophy Progression Prediction
Source: Transl Vis Sci Technol. 2024 Aug 5;13(8):6. doi: 10.1167/tvst.13.8.6 (PMC11309039; doi:10.1167/tvst.13.8.6)

SUPPLEMENTARY TABLES AND FIGURES

Supplementary Table 1. Randomly Generated Sets of Hyperparameters Used for Training the VGG16 Network

Hyperparameter	Search Space
Transformations	"Moderate": RandomHorizontalFlip (p= 0.5); RandomVerticalFlip(p=0.5); RandomRotation(10, interpolation=InterpolationMode.BILINEAR, expand = False), RandomAffine(5, translate=(0.05, 0.05), shear=5, interpolation=InterpolationMode.BILINEAR)<br>"Complex": RandomHorizontalFlip (p= 0.5); RandomVerticalFlip(p=0.5); RandomAffine(10, translate=(0.05, 0.05), shear=5, interpolation=InterpolationMode.BILINEAR), ColorJitter(brightness=0.3, contrast = 0.5, saturation = 0.5, hue = 0.5)
Epochs	50, 65, 80
Learning rate	5e-5, 1e-4, 2.5e-4, 5e-4, 7.5e-4, 1e-3, 2.5e-3, 5e-3, 7.5e-3, 1e-2
Weight decay	1e-2, 5e-3, 1e-3, 5e-4, 1e-4
Dropout	0, 0.1, 0.2, 0.3, 0.4, 0.5, 0.6, 0.7

Supplementary Table 2. Fixed Hyperparameter Choices Used for Training the VGG16 Network

Hyperparameter	Fixed Choice
Optimizer	Adam
Scheduler	OneCycle
Loss	MSE
Model architecture	VGG16

Supplementary Figure 1. Representative randomly chosen fundus autofluorescence images next to their mask output and Convex Hull.

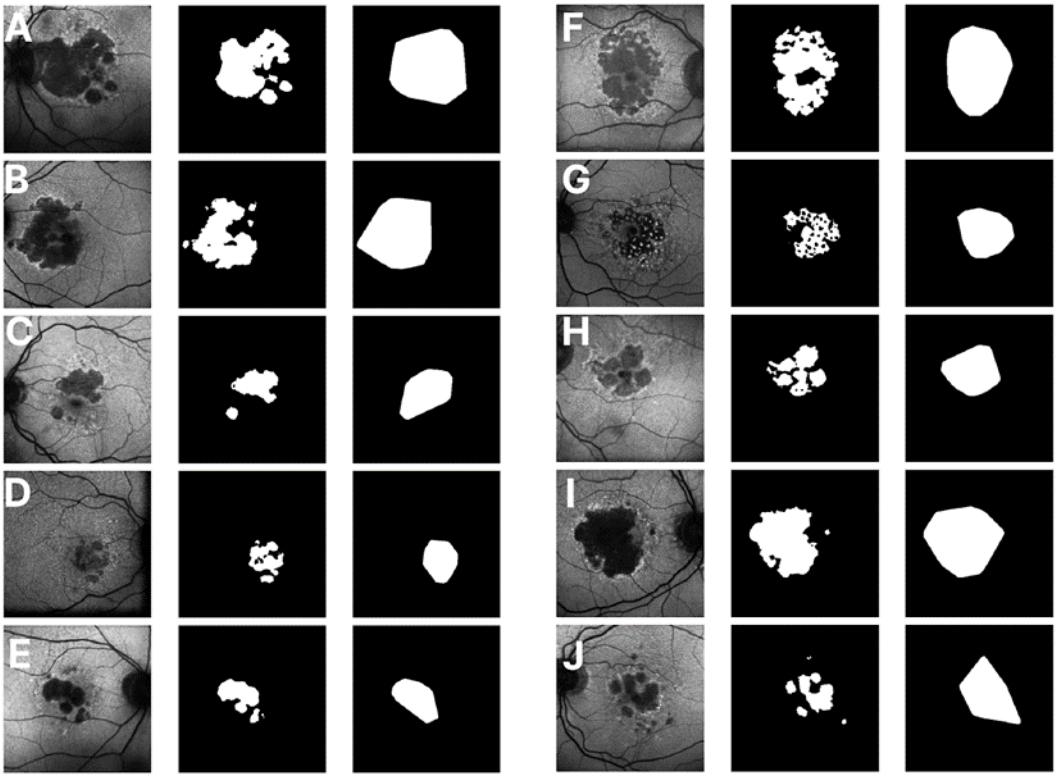

Supplement: Supplement 1 [file tvst-13-8-6_s001.pdf]
